# Obituary

**Published:** 1855-10

**Authors:** 


*Obituary.*—M. Valleix, the distinguished physician to La Pitie, at Paris, died on the 13th of July, aged 48 years.

Mr. Andrew Crosse, the well known natural philosopher, whose alleged

*creation* of the *Acarus Crossei* made such a tumult among the physiologists, a few years since, died recently at his seat in Somersetshire, England.

Died at Saratoga, on the 20th of August, Dr. Moreton Stillé, of Philadelphia, æt. 33. Dr. Stillé had just prepared for the press a work on medical jurisprudence, which is highly spoken of by those who have had access to it. It will be issued immediately, we presume under the editorial supervision of his distinguished brother, Dr. Alfred Stillé.

